# Participation of Emerging Commercial Farmers in the Strategic Private-Sector Investment Interventions

**DOI:** 10.3390/agriculture15050450

**Published:** 2025-02-20

**Authors:** Sandile Jason Mnikathi, Simphiwe Innocentia Hlatshwayo, Ojo Temitope, Mjabuliseni Simon Cloapas Ngidi

**Affiliations:** 1 Department of Agricultural Extension and Rural Resource Management, School of Agricultural, Earth and Environmental Sciences, College of Agriculture, Engineering and Science, University of KwaZulu-Natal, Private Bag X01, Scottsville, Pietermaritzburg 3201, South Africa; 2 African Centre for Food Security, School of Agricultural, Earth and Environmental Sciences, College of Agriculture, Engineering and Science, University of KwaZulu-Natal, Private Bag X01, Scottsville, Pietermaritzburg 3201, South Africa; 3 Centre for Transformative Agricultural and Food Systems, School of Agricultural, Earth and Environmental Sciences, College of Agriculture, Engineering and Science, University of KwaZulu-Natal, Private Bag X01, Scottsville, Pietermaritzburg 3201, South Africa; 4 Department of Agricultural Economics, Obafemi Awolowo University, Ile-Ife 220103, Nigeria; 5 Department of Plant, Food and Environmental Sciences, Faculty of Agriculture, Dalhousie University, Truro, NS B2N 5E3, Canada

**Keywords:** emerging farmers, commercial farmers, investment intervention, double-hurdle model, South Africa

## Abstract

Private sector investment interventions serve as essential mechanisms for creating efficient, cost-effective financial solutions and technological support for emerging farmers in developing economies, yet their successful implementation is influenced by various contextual and socioeconomic factors. Using a quantitative research approach, this study examined the factors influencing participation in private sector investment interventions among 121 emerging commercial farmers in KwaZulu-Natal, South Africa, utilizing a Poisson regression model to analyze four key intervention areas: credit access, market access, technical support, and spot supply. The first-hurdle model revealed that age and training skills negatively influenced market access while the training period showed positive influence, and similarly, the second-hurdle equation demonstrated that employment status and training period positively influenced participation intensity levels, though age maintained its negative impact. The findings of the first-hurdle model reveal that age and training skills negatively influenced market participation. The study concludes that employment status and training period positively impacted technical support adoption, with household size and training period emerging as significant determinants of intervention success. The private sector needs to develop strategic partnership models that encourage emerging farmers to participate intensively in interventions that are designed to improve their production and productivity. There is a need for targeted capacity-building programmes and enhanced extension services to improve emerging commercial farmers’ participation in private-sector initiatives.

## 1 Introduction

In most developing economies, agriculture is a significant contributor to gross domestic product (GDP), foreign exchange, export earnings, employment, and capital generation [1,2]. It plays a vital role in the economic growth and poverty alleviation in developing countries [3,4]. The sector is one of the key drivers of South Africa’s economies [5]. According to a report from the South African News Agency (2023), the number of people employed in the agricultural sector increased by 6.6% in the first quarter of 2022, from 792,000 people in the first quarter of 2021 to 844,000 people in the same quarter of 2022. South African agriculture is characterized by “two agricultures” with one being highly capitalized and large-scale, and the other comprised of black smallholders who mostly resides in the former homeland areas [6]. The latter include emerging commercial farmers who produce to generate profit through market sales and have access to capital through loans, grants, or personal savings. Neves [7] reported that emerging commercial farmers are those farmers involved in bulk commodities agricultural production. Unlike small-scale farmers, whose production is within 0.1 ha to 20 ha with land for production allocated by tribal authority, emerging commercial farmers produce on the farm scale of more than 20 ha, with land ownership registered, with title deed, and have clear cadastral boundaries. These farmers play an important role in economic growth; they significantly contribute towards employment creation and income generation, especially in rural areas. Despite the recognition of their significance, emerging farmers are often constrained by a multitude of factors that hinders their participation in most formal sectors. The integration of emerging farmers into private-sector investment interventions can help to enhance productivity and promote inclusive agricultural transformation. Furthermore, private-sector investment in agriculture has been recognized as a crucial catalyst for agricultural transformation in developing countries. According to Otsuka and Fan [8], strategic private-sector partnerships can bridge critical gaps in agricultural value chains, particularly in areas of technology transfer, market access, and financial services for emerging farmers.

Private-sector investment interventions include a wide range of initiatives, including financial support, technical assistance, market linkages, and infrastructure development, aimed at enhancing the productivity, profitability, and sustainability of agricultural enterprises [9]. These interventions are often led by private investors, agribusinesses, development finance institutions, and non-governmental organizations (NGOs). The private-sector investment interventions create more efficient, cost-effective, and customized financial solutions to unlock credit and manage risk for emerging farmers [10]. The interventions introduce emerging farmers to user-friendly information and communications technology (ICT) applications and capacity-building services that provide yield-enhancing solutions [11]. The private-sector partnership provides opportunities for participation in the profitable value chain. In the vertical value chain, the interventions provide post-harvest services that offer processing and packaging solutions to increase shelf life, and storage solutions that target different types of agricultural produce. Additionally, private-sector investment intervention offers direct-to-market for the farmers. These strategic models remove or reduce middlemen from the distribution chain and multi-stakeholder platforms that facilitate information flows and business transactions between suppliers and buyers [12]. Despite the potential benefits of private sector investment interventions, the participation of emerging farmers in these initiatives is influenced by various contextual, institutional, socioeconomic, and individual factors.

Agricultural production in South Africa and elsewhere in the world is faced with numerous challenges. These challenges are influenced by both internal and external environmental factors. The unpredictable climate and weather changes, inadequate inputs, and poor infrastructures are well-documented factors that affects both well-developed and underdeveloped farmers. In South Africa, emerging commercial farmers are further impacted by the lack of or inappropriate and inefficient flow of farming information, insufficient market information, shortage of finance and credit facilities, unlawful middlemen, inadequate access to advanced technology, limited access to affordable finances, market and unfair market conditions, high production and transport costs, and low skills [2,11,13]. These challenges further exacerbate lower bound productivity, low life expectancy, food insecurity, undernourishment, high infant mortality, crime in society, and rural–urban migration [2]. The challenges require multiple research studies and practical interventions to be undertaken so that urgent matters are ironed out sequentially and subsequently eliminated. Additionally, Mabaya and Cramer [14] highlight that emerging commercial farmers in Sub-Saharan Africa often face structural constraints in accessing private-sector investment opportunities, with only 23% of such farmers successfully integrating into formal agribusiness value chains, despite the potential for increased productivity and income generation.

Understanding the determinants of emerging farmers’ participation in private-sector investment interventions is essential for promoting inclusive and sustainable agricultural development. The determinants such as age, gender, employment status, household size, and farm association are expected to impact the probability of private-sector investment interventions adoption and the intensity by the emerging commercial farmers in this study. By identifying and addressing the barriers that hinder participation, policymakers and development practitioners can devise targeted strategies to enhance the effectiveness and inclusivity of private-sector-led initiatives. There are few to no studies that have been conducted on the factors that influence the participation of emerging commercial farmers in private-sector investment interventions, especially in the South African context. Several studies [13–16] were conducted on the impact of private-sector investment interventions on the rural economy. However, they did not investigate the factors that influence the participation of emerging commercial farmers in the private sector. Also, most of them are conducted in other parts of Africa, but not South Africa. This study seeks to identify the key factors that influence the participation of emerging farmers in private-sector investment interventions. It also aims to examine the interplay between these factors and the intensity level of participation of these farmers in the private-sector interventions. This research is expected to contribute to the academic literature on agricultural development, private-sector engagement, and rural transformation. Furthermore, the study will have a significant contribution in providing recommendations for policymakers, development practitioners, and other stakeholders to enhance the participation of emerging farmers in private-sector-led initiatives. The study structure consists of four sections, including this introductory section. The second section discusses the methodology used for this study, which includes a description of the study area, Participants’ selection and Data collection method, and Data analysis method. The third section summarizes and discusses the research findings, focusing on two key themes: Drivers of participation of emerging farmers in the private sector investment interventions and Determinants of the Intensity of private sector investment intervention Adoption. Finally, the conclusion provides a summary and critique of the findings and areas for future research.

## 2 Methodology

### 2.1 Description of the Study Area

**Figure 1:**
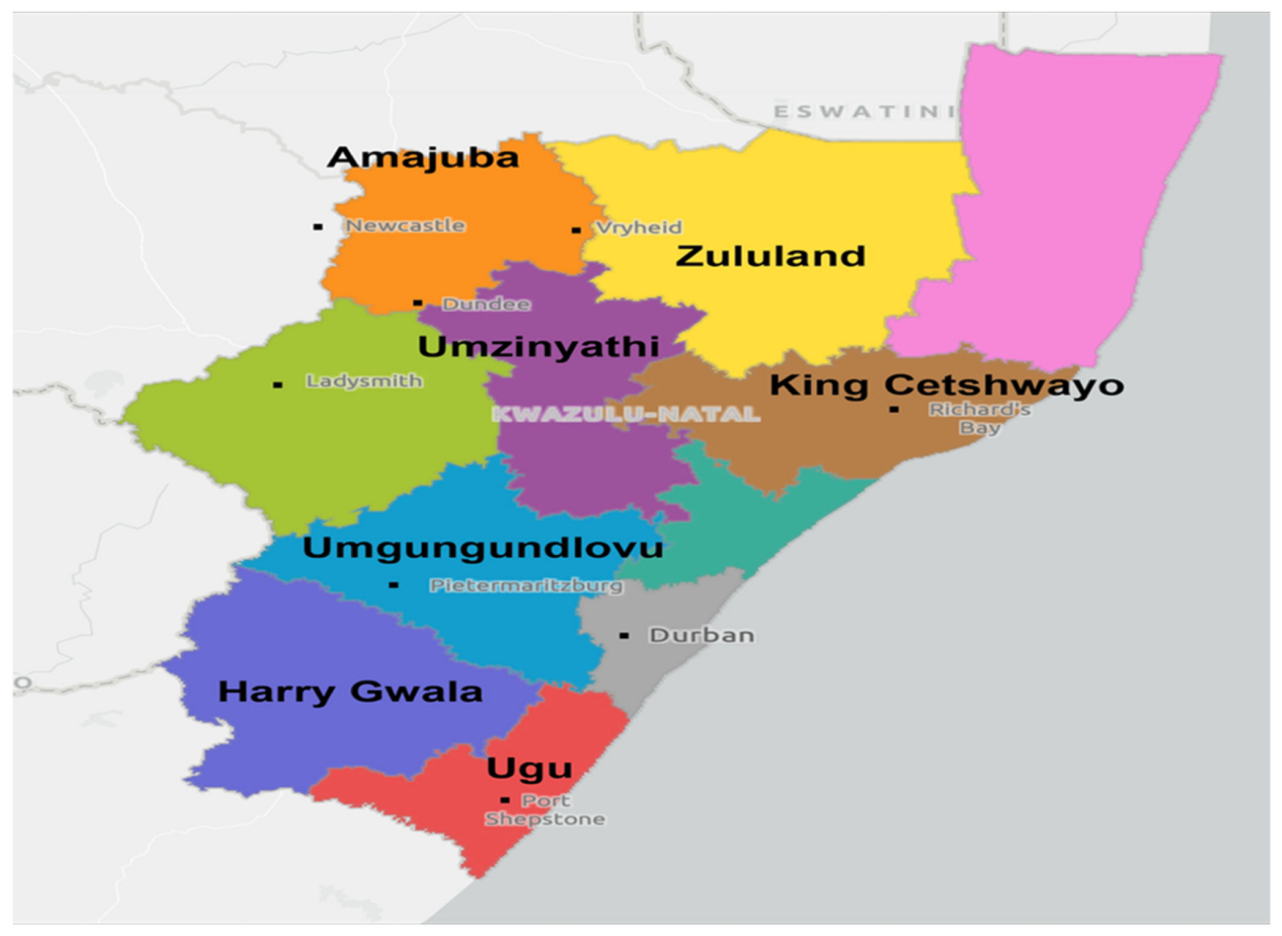
Map of the municipalities in KwaZulu-Natal province of South Africa, with all municipalities named and district municipalities shaded different colours (2023).

The research was conducted in KwaZulu-Natal province and was refined to seven districts of the province. This was due to the presence of emerging Black commercial farmers who are in partnership with the private sector. The districts that the study included were Harry Gwala district, uGu District, uThungulu district, uMgungundlovu district, uMzinyathi district, Zululand, and King Cetshwayo district (Figure 1). These districts were chosen as the area of study because they are occupied by emerging farmers who are involved in forestry or timber production. The majority of the emerging commercial farmers are involved in the production of sugarcane and forestry as land reform claimants [17]. The were chosen as the area of study because they are occupied by emerging farmers who are involved in forestry or timber production. The majority of the emerging commercial farmers who are involved in forestry or timber production. The majority of the emerging commercial farmers are involved in the production of sugarcane and forestry as land reform claimants [17]. The The districts are known for its progressive farming. The two largest forest owners in South Africa, Sappi and Mondi, are found in KZN but operate across Mpumalanga and Eastern Cape. The province is the country’s leading producer of timber where timber production accounts for 6.5% of KZN’s agricultural output (KZN DEDTEA, 2016). The province receives more than 1000 mm of rain each year, most of it falling during summer, which together with its fertile soils creates productive conditions that make agriculture central to the economy. Agriculture in the province is extremely diverse and varies according to patterns of its topography.

### 2.2 Participants’ Selection and Data-Collection Method

The Department of Forestry, Fisheries and the Environment through the KwaZulu Natal province office was provided the database of emerging commercial farmers that benefited from the private-sector investment interventions. The list obtained from the benefited from the private-sector investment interventions. The list obtained from the Department included the contact details of the emerging commercial farmers contracted to partment included the contact details of the emerging commercial farmers contracted to the private sector. The emerging commercial farmers were selected on the condition that, firstly, they had been in a strategic partnership over a 12-month period. Secondly, within the same period, there has been some form of an investment by a strategic partner that also included the market access. Respondents were either the farm owner or individual responsible for farm overall management and operations. While more than 250 emerging farmers were found in the sampling frame, only 135 met the criteria of having 20 ha farm size. Of the 135 emerging farmers that met the criteria for selection, about 121 were selected to be interviewed.

This study used a quantitative research method, utilising questionnaires to collect data across eight of the 11 districts in KwaZulu Natal. A simple random sampling technique was implemented for participant selection, ensuring each participant member had an equal probability of inclusion—a method widely recognised as the most straightforward among probability sampling approaches [18,19]. Data collection was conducted through face-to-face interviews, with questionnaires administered in isiZulu to enhance response rates and participant comprehension. The research instrument encompassed multiple domains: demographic information, contract participation and capacity building, productivity inputs, marketing and credit access, income status, and food security indicators. This comprehensive approach facilitated an in-depth examination of participants’ understanding and attitudes toward partnership benefits, while also capturing farmers’ perceptions of success in their agricultural endeavors.

### 2.3 Data Analysis Method

The analytical framework encompassed both descriptive and econometric methodologies, utilizing SPSS (version 27) and STATA (version 11) statistical software packages for data processing and analysis. Prior to conducting the main analysis, comprehensive data quality checks and reliability tests were performed. These included data profiling, validation procedures, and various statistical tests including normality assessments to check for distribution patterns, outlier detection, and correlation analyses. Descriptive statistical measures, including means, percentages, frequencies, and standard deviations, were employed to characterize the sociodemographic attributes of the sampled participants. To examine the participation levels of emerging commercial farmers in private-sector investment interventions and the corresponding intensity of adoption, the study implemented a Poisson regression model. This analytical approach was selected due to its appropriateness for count data, as both participation and adoption intensity were quantified using positive integer values. The Poisson regression framework provided a robust methodological foundation for analysing these discrete numerical outcomes, enabling a comprehensive assessment of intervention engagement patterns.

The Poisson distribution assumes Equi dispersion, meaning equality between the variance and the mean. Under-dispersion occurs when the variance found is smaller than the mean, while over-dispersion occurs when the variance exceeds the mean [20–22]. Using the approach from Abate and Addis [23], let y_i_ be the number of occurrences at a given time or exposure period, represented at a rate of *µ*_i_. The below equations represent the specifications for the Poisson regression model:

(1) $$\text{P}\left({\text{Y}}_{\text{i}}={\text{y}}_{\text{i}},\mu \right)=\frac{{\text{e}}^{-\mu_{\text{i}}}\mu^{\text{y}}}{{\text{y}}_{\text{i}}^{!}},\mu_{\text{i}}>0,i=1,2\ldots n,{\text{ and y}}_{\text{i}}=0,1,2,3\ldots$$

The equation can be further represented by:

(2) $$\mu =\exp\left(\beta_{0}+\beta_{1}{\text{x}}_{1\text{i}}+\beta_{2}{\text{x}}_{2\text{i}}+\ldots +\beta_{\text{k}}{\text{x}}_{\text{ki}}\right)$$

In this study, y_i_ represents the value of an event count outcome variable with a mean parameter of *µ*_i_ that occurs during a specific time or exposure period [23]. Assumed to be a non-linear function of the independent variables, *µ*_i_ represents the mean and variance of the Poisson distribution. β0 is the intercept of the model, where the coefficients of independent variables are denoted as β_1_; β_2_ … β_k_ and the quantity of explanatory variables is K [24].

## 3 Results and Discussion

### 3.1 Descriptive Results

Table 1 presents the descriptive statistics of sociodemographic characteristics among emerging commercial farmers in the study area. The analysis revealed universal participation (100%) in forestry production among respondents. Gender distribution indicated male predominance at 64%, compared to 36% female representation, reflecting the characteristic male-dominated nature of forestry farming in developing nations, including South Africa. The sample respondents exhibited a mean age of 56.20 years, with a significant majority (86%) reporting marital status as married, while only 1% identified as single. Educational attainment analysis revealed that 23% of respondents had completed primary education, 22% had attained tertiary education (university or college level), and 17% reported no formal education, indicating that approximately half of the sample population had received formal education. Employment status data showed that 46% of respondents were engaged in farm-based employment, while 11% reported unemployment. Regarding private sector investment intervention accessibility, 41% of emerging farmers reported credit access, 37% indicated market access, and a notably smaller proportion (6%) received technical support. The emerging farmers demonstrated substantial agricultural experience, with a mean of 16.54 years in farming activities. Furthermore, the average household size among emerging farmers was 8.25 members, suggesting adequate family labor availability, which is particularly relevant given the sector’s reliance on familial workforce contributions.

### 3.2 Empirical Results

Table 2 delineates the determinant factors influencing emerging farmers’ participation in private-sector investment interventions, specifically examining four key domains: credit access, market access, technical support, and spot supply mechanisms. The empirical analysis revealed a significant negative correlation between farmers’ age and market access propensity. This inverse relationship suggests that advancing age corresponds with diminished market participation and reduced engagement in spot supply activities. This phenomenon can be attributed to increased risk aversion among older agriculturalists, who tend to prioritize subsistence production over commercial endeavors. These findings diverge from previous research by Mdlalose [25] and Agholor et al. [26], who identified a positive and significant relationship between respondent age and market participation. Their studies posited that younger agricultural practitioners demonstrate a greater inclination toward risk-taking and market strategy adoption. Furthermore, they postulated that recent entrants to the agricultural sector, predominantly younger farmers, exhibit heightened motivation to implement marketing strategies to facilitate farm enterprise growth.

The results showed that educational level of emerging farmers had a negative and significant impact on the market access at a 10% level. This means that most of the emerging farmers did not receive formal education which made them not to understand how credit scheme works and their terms and conditions. The results were in line with Zulu et al. [27], who found a negative and significant relationship between level of education and access to credit. The authors explained that their results mean that educated farmers have access to other forms of income to finance their farm production. On the contrary, Assogba et al. [28], Szymkowiak et al. [29], and Haryanto et al. [30] found that educational level had a positive effect on access to credit financial services. These studies explained that educated farmers have the ability to understand the credit terms and conditions and also, they have more advantages to have secured collateral, which allows them to access credit.

Technical support in this study refers to a support that help emerging farmers to acquire specialized service or skill that they do not have in the farm. These technical supports are needed in order to operate more effectively and reinforce sustainability. The technical support is more of the extension services that are provided by the government to emerging farmers. The results showed that employment status had a positive and significant (*p* < 0.1) influence on technical support and spot supply. This implies that employed farmers were utilizing the technical support that they received from extension officers and increased their spot supply. These results were supported by Taylor and Bhasme [31] and Takahashi et al. [32], who found that extension services such as technology adoption, technical support, and networks have a positive impact on emerging farmers. These studies stated that agricultural extension services help towards knowledge diffusion and innovation to emerging farmers and also assist farmers to adopt new best practices or technologies that help them to produce efficiently.

Land tenure had a positive and significant influence on the technical support. This means that emerging farmers who owned land and had property rights benefited from technical support. This is because emerging farmers with land utilize the resources they have and that have been provided to them in order to increase farm production. The results were supported by Hailu et al. [33], who reported that to most farmers, owning an arable land is set as a prerequisite to adopt and employ technical support such as agricultural technologies. The authors further explained that farmers who own land want to employ technologies where the final crop yield could not be shared and subdivided. Singha et al. [34] found that size of operational land holding had a positively significant relationship with respect to technology adoption of rice cultivation and dairy farming. The study explained that farmers who owned larger operational farm size tended to adopt more technology.

The results of this study also showed a positive and significant relationship between household size and spot supply among emerging farmers. This means that as the household size of emerging farmers increases, their spot supply increases. Spot supply refers to the traditional means of price transmission in agriculture, which developed around generic or perishable products produced on many farms of similar size but geographically dispersed [35]. It allows farmers to sell to buyers (wholesalers, processors, brokers, and shippers) at an immediate transaction and delivery. Having more family members in the household results in sharing of duties in the farm, meaning that some household members were involved in supplying commodities that are available for immediate delivery.

The training period showed a positive and significant influence on credit access, market access, and technical support among emerging farmers. This implies that as the training period increased, the technical support that emerging farmers received also increased, and they were able to access credit and produce more so that they could participate in the market. This is because when emerging farmers are trained well, they became more interested in adopting any new technology and they produce more to be able to participate in the formal market. Opolot et al. [36] confirmed these results by reporting that increased training allows farmers to improve their existing skills while they acquire new skills and knowledge for improved competitiveness. De Lauwere et al. [37] also attested that the increased training period enabled farmers to obtain competences in value adding and marketing, which is important in making farming more profitable.

The results showed that being satisfied with partnership had a positive and significant (*p* < 0.1) influence on access to market. This means that the satisfaction of emerging farmers with their partners resulted in them being able to access credit. The possible plausible explanation to this is that emerging farmers were receiving help from their partners on the required information and terms and conditions of acquiring credit. The results were in line with Ponnusamy et al. [38] and Zhou et al. [39], who found a positive correlation between partnership and access to credit. These studies explained that partnership projects empowered farmers with interaction meetings where farmers understood the innovative ways of accessing financial resources, which also increased their confidence level. These studies further explained that partnership project help farmers to understand financial security and collaterals that are required by the bank.

Training skills received by emerging farmers had a negative and significant impact on market access. This means that emerging farmers did not receive enough training on market-related issues, which resulted to them not participating in the market. The results were in line with that of the Food and Agriculture Organization [40]; Hlatshwayo et al. [41] found that extension services such as training had a negative and significant impact on farmers’ participation in the market. These studies reported that sometimes farmers do not receive enough training and end up utilizing whatever resources they have to produce only for consumption. They further stated that agricultural extension officers are understaffed in South Africa, and they do not have enough market competencies. In contrary, Kyaw et al. [42] and Zondi et al. [43] found a positive and significant relationship between training skills received by farmers and market participation. These studies’ findings reported that extension services such as training provide advice and information on new technologies that increase farmers’ production and are able to access the market. They also stated that extension officers train farmers with agricultural skills, intelligence, and knowledge to facilitate market access and trade.

The effect of private-sector investment interventions on emerging farmers.

The study recognized that the participation of emerging farmers on private-sector investment intervention is affected by many factors, which include socioeconomic, production, and external factors that were heterogeneous and could result in self-selection bias. Therefore, holding other factors constant, the study assessed the effect that the four private-sector investment interventions had on emerging farmers’ participation (Table 3). The results showed that all the four interventions (credit access, market access, technical support, and spot supply) had a positive and significant effect on participation of emerging farmers in the private interventions.

### 3.3 Determinants of the Intensity of Private-Sector Investment Intervention Adoption

The results of the intensity of private-sector investment intervention adoption are presented in Table 4. As shown in Table 4, the estimation of Akaike information criterion (AIC) and Bayesian information criterion (BIC) are essential to indicate a better model in analyzing count data of the level of adoption of private-sector interventions. In this study, the Poisson regression model was used, the AIC value was 493.658, and the BIC value was 530.003. Age of household head, employment status, and training period were the main factors that significantly affected the intensity of private investment intervention adoption by emerging farmers in KwaZulu Natal. The results showed that age negatively influenced adoption of private-sector investment intervention by emerging farmers, and it was significant. This means that as age of emerging farmers increases, their level of adoption decreases. As it was mentioned before, when farmers get older, they become more risk adverse and mainly produce for their family survival rather than selling, therefore they become skeptical to participate in any interventions. The results were in contrary with Sebatta [44] and Nwafor [45], who found a positive and significant relationship between age of farmers and adoption of one of the private-sector investment interventions, which is access to market. These studies reported that older farmers tend to make better decisions, and they have established relationships and partnerships over the years that enable them to access the formal market. They also stated that older people often use their retirement funds to invest in farming and produce more.

The results showed that employment status had a positive and significant (*p* < 0.1) influence on the intensity of private-sector investment intervention. This means that as emerging farmers get employed, their adoption of private-sector investment interventions increases. This is because farmers who are employed, they use their income to invest more in farming and be able to access credit, utilize the support they receive from government, and be able to participate in the market. These results were supported by Kiplimo et al. [46] and Zulu et al. [27], who found that employment status had a positive and significant effect on adoption of Information Communication Technology and access to credit. These studies reported that farmers who are employed are able to generate enough income outside the farm and accrue more assets that can be used as security when seeking credit services. Lastly, training period showed a positive and significant influence on the level of adoption of private-sector investment interventions. This means that as the training period increased, the level of adoption increased among emerging farmers. These results were confirmed by De Lauwere et al. [37] and Opolot et al. [36], who explained that when the training period increases it allows farmers to master the skills that they possess and obtain more new skills, which allows them to produce more effectively and participate in all the formal interventions.

## 4 Conclusions and Recommendations

Enhancing emerging commercial farmers’ engagement in private-sector investment interventions carries significant implications for agricultural productivity and marketing capabilities, ultimately contributing to improved food and nutrition security outcomes. This empirical investigation identifies critical determinants affecting emerging commercial farmers’ participation in private-sector investment mechanisms. The findings demonstrate that participation patterns are influenced by multiple variables: age, educational attainment, employment status, land tenure arrangements, household composition, training duration, partnership satisfaction, and acquired technical skills.

The analysis reveals complex relationships among these variables across different intervention domains. Market access exhibited negative correlations with age and training skills, while training duration demonstrated an inverse relationship. In the context of credit access, educational attainment showed a negative association, whereas training duration and partnership satisfaction displayed positive correlations. Technical support participation was positively influenced by employment status, land tenure arrangements, and training duration. Regarding spot supply engagement, household size and employment status demonstrated significant positive associations, while age exhibited a significant negative relationship.

Three key factors significantly influenced the intensity of participation: age demonstrated a negative correlation, while employment status and training duration showed positive associations. The study concludes that employment status and training period positively impacted technical support adoption, with household size and training period emerging as significant determinants of intervention success. The study recommends that the private sector needs to develop strategic partnership models that encourage emerging farmers to participate intensively in interventions that are designed to improve their production and productivity. There is a need for targeted capacity-building programmes and enhanced extension services to improve emerging commercial farmers’ participation in private-sector initiatives.

The findings of this study are based on data collected in the KwaZulu-Natal province of South Africa and may not be applicable as a general representation of emerging commercial farmers on a national or international scale. There is a need for similar studies to be conducted across all nine provinces of South Africa. This study specifically examined the impact of private-sector strategic interventions on emerging commercial farmers. However, future research could expand to include other types of farmers, such as smallholder and subsistence farmers.

## Figures and Tables

**Table 1:** Sociodemographic characteristics of emerging farmers.

Qualitative Variables	Frequency	Percentage
Agricultural commercial activity		
Forestry	121	100
Timber	0	0
Gender		
Male	77	64
Female	44	36
Educational level		
Primary school	28	23
Matric	20	17
Higher vocational school	25	21
University/ college	27	22
None	21	17
Marital status		
Married	104	86
Divorced	9	7
Single	1	1
Living together like husbands and wife	7	6
Employment status		
Self-employed	26	22
Employed privately	25	21
Employed at the farm	56	46
Not employed	14	11
Private sector investment interventions		
Market access	45	37
Technical support	7	6
Credit access	50	41
Spot supply	19	16
Quantitative variables	Mean	Standard deviation
Age	56.20	8.436
Farming experience	16.54	5.005
Household size	8.25	2.504

**Table 2:** Drivers of participation of emerging farmers in the private-sector investment interventions. Poisson regression model.

Variables	Credit Access			Market Access			Technical Support			Spot Supply		
	Coefficient	Std. errs.	*p*-Value	Coefficient	Std. errs.	*p*-Value	Coefficient	Std. errs.	*p*-Value	Coefficient	Std. errs.	*p*-Value
Age	−0.00111	0.005625	0.844	−0.00944	0.005567	0.09 *	−0.00667	0.005655	0.238	−0.01188	0.005492	0.789
Gender	0.066845	0.096266	0.487	0.0225	0.095236	0.813	0.036521	0.096793	0.706	0.035573	0.094417	0.706
Education	−0.05543	0.032333	0.086 *	−0.04352	0.032036	0.174	0.011451	0.032566	0.725	−0.0297	0.031615	0.347
Marital Status	−0.05468	0.060843	0.369	−0.04775	0.060952	0.433	−0.08678	0.062293	0.164	−0.0274	0.059647	0.646
Employment status	0.061464	0.048775	0.208	0.033388	0.048376	0.49	0.080715	0.049185	0.076*	0.163696	0.047618	0.001 ***
Farming period	0.005203	0.009689	0.591	0.010692	0.0096	0.265	−0.00852	0.009759	0.383	0.000322	0.009483	0.973
Farm size	−5.9 × 10^−5^	4.62 × 10^−5^	0.206	−3.6 × 10^−5^	0.000046	0.435	−2 × 10^−5^	4.69 × 10^−5^	0.678	5.72 x 10^−5^	4.53 × 10^−5^	0.207
Land tenure	0.003567	0.105672	0.816	0.009148	0.014458	0.527	0.030596	0.017736	0.085 *	0.335367	0.048651	0.876
Household size	0.004527	0.019202	0.814	0.010714	0.01905	0.574	0.021174	0.019351	0.274	0.255101	0.018628	0.001 **
Farm Association	−0.06139	0.106968	0.566	−0.03664	0.112835	0.745	0.058552	0.117749	0.619	−0.04253	0.104646	0.684
Training Period	0.126954	0.047138	0.007 ***	0.126323	0.046869	0.007 ***	0.103113	0.047703	0.031**	0.038142	0.045997	0.407
Satisfied with partnership	0.078427	0.047498	0.099 *	0.07055	0.049572	0.155	0.060844	0.047589	0.201	0.076557	0.045679	0.569
Training skills received	−0.02283	0.01435	0.112	−0.0368	0.014425	0.011 **	−0.01286	0.01467	0.381	−0.01261	0.01351	0.351
Extension office visit	−0.06258	0.076929	0.416	−0.04158	0.076205	0.585	0.038247	0.077497	0.622	−0.02697	0.075449	0.721
_cons	0.392737	0.553271	0.478	0.68015	0.56006	0.225	−0.01608	0.572488	0.978	1.075703	0.527605	0.041 **

*** *p* < 0.001,

** *p* < 0.05,

* *p* < 0.1.

**Table 3:** The effect of private-sector investment interventions on emerging farmers.

Equation	Obs	Params	RMSE	R-Squared	chi2	*p* > chi2
Credit access	121	13	0.464108	0.125	18.9	0.047 **
Market access	121	14	0.460709	0.1463	21.55	0.084 *
technical	121	14	0.467008	0.1174	17.49	0.038 **
Spot supply	121	12	0.455155	0.0957	12.8	0.087 *

** *p* < 0.05,

* *p* < 0.1.

**Table 4:** The determinants of the intensity of private-sector investment interventions adoption.

Variables	Poisson			Marginal Effect		
	Coefficient	Std. Errs.	*p*-Value	Dy/dx	Std. Errs.	*p*-Value
Age	−0.013	0.008	0.092 *	−0.029	0.017	0.090 *
Gender	0.085	0.138	0.538	0.179	0.291	0.538
Education	−0.066	0.045	0.143	−0.140	0.095	0.141
Marital Status	−0.143	0.095	0.132	−0.302	0.200	0.130
Employment status	0.114	0.069	0.097 *	0.241	0.145	0.095 *
Farming period	0.002	0.014	0.900	0.004	0.030	0.900
Commercial Area size	0.000	0.000	0.249	−0.001	0.000	0.244
Land tenure system	0.043	0.029	0.135	0.090	0.060	0.134
Household size	0.041	0.027	0.127	0.087	0.057	0.125
Training Period	0.189	0.070	0.007 ***	0.400	0.147	0.006 ***
Training skills required	−0.027	0.021	0.186	−0.058	0.043	0.184
Involvement Improvement	0.121	0.149	0.414	0.257	0.314	0.413
Constant	0.314	0.696	0.652			
Mean dependent variable	2.231	SD dependent var	1.783			
Pseudo r-squared	0.052	Number of obs	121.000			
Chi-square	25.729	Prob > chi2	0.012			
Akaike crit. (AIC)	493.658	Bayesian crit. (BIC)	530.003			

*** *p* < 0.001,

* *p* < 0.1.

## Data Availability

Data are available on request from the authors.
